# Total sacrectomy with a combined antero-posterior surgical approach for malignant sacral tumours

**DOI:** 10.1007/s00264-021-05006-4

**Published:** 2021-03-25

**Authors:** Feifei Pu, Zhicai Zhang, Baichuan Wang, Qiang Wu, Jianxiang Liu, Zengwu Shao

**Affiliations:** Department of Orthopedics, Union Hospital, Tongji Medical College, Huazhong University of Science and Technology, 1277 Jiefang Avenue, Wuhan, 430022 Hubei China

**Keywords:** Sacral tumour, Bone tumour, Total sacrectomy, Spinopelvic reconstruction

## Abstract

**Purpose:**

To investigate the indications, approaches, resection methods, and complications of total sacrectomy with a combined antero-posterior approach for malignant sacral tumours.

**Methods:**

Fourteen cases of primary malignant sacral tumours treated with total sacrectomy between January 2012 and 2018 were retrospectively analysed. All patients presented with pre-operative lumbosacral pain or constipation. A combined antero-posterior approach was used for tumour resection, and the spinal pedicle screw rod system was used to achieve ilio-lumbar stability. The visual analogue scale (VAS) and Musculoskeletal Tumor Society (MSTS) scores were used to assess pain and lower limb function, respectively. The mean operative time and intra-operative blood loss were 6.54 hours and 2935 mL, respectively. The mean follow-up period was 62 months.

**Results:**

None of the patients died peri-operatively. At the last follow-up, ten patients were continuously disease-free, three were alive with disease, and one died of disease from lung metastasis. Tumour recurrence occurred in three patients. The MSTS scores ranged from 6 to 28 (20.00–93.33%, 6/30–28/30) with an average of 20 (66.67%, 20/30). Seven patients could walk independently in public, five could only walk at home using a walking aid, and two could only lie down and stand for a short time. Thirteen patients developed post-operative complications such as skin necrosis, screw loosening, connecting rod fracture, neuropathic pain, sciatic nerve injury, dysuria, and urinary incontinence.

**Conclusion:**

Total sacrectomy can effectively treat malignant sacral tumours with good resection boundaries and prognosis. However, the high incidence of post-operative complications may impact post-operative neurological function.

## Introduction

Sacral tumours are rare and account for only 1.0–3.5% of primary bone tumours [1, 2]. The onset of sacral tumours is relatively insidious, with no specific initial symptoms or characteristic auxiliary examination findings [3]. These tumours can be resistant to radio- and chemotherapy, and even if chemotherapy is effective, very few patients can be completely cured without surgical excision [4]. Total sacrectomy is a radical but effective treatment, and only extensive resection of the tumour can control local recurrence and prolong survival time [5]. Selection of the appropriate surgical approach is vital to resect sacral tumours completely, and most scholars adopt the combined antero-posterior approach [6]. This approach safely separates the rectum and blood vessels in the anterior sacral region, and the posterior incision is closed with a rectus flap [7, 8]. However, total sacrectomy can destroy the continuity of the sacroiliac joint; hence, post-resection restoration of stress conduction and stability of the lumbosacral region directly determines the patient’s postoperative function and quality of life [9, 10]. This study retrospectively investigated the surgical indications, approaches, resection methods, and complications of total sacrectomy with a combined antero-posterior surgical approach in malignant sacral tumours.

## Patients and methods

### Patients

From January 2012 to January 2018, 14 patients (eight male and six female; mean age, 41.21 years [14–63 years]) with primary malignant sacral tumours were treated with total sacrectomy with ilio-lumbar reconstruction via an antero-posterior surgical approach. The tumours were located in the S1–S2 regions in three cases, S1–S3 in five, S1–S4 in four, and S1–S5 in two. A biopsy can be performed using a percutaneous needle to confirm both the diagnosis and grade of the bone tumour, as well as direct treatment. However, because the biopsy tract and the skin can become contaminated with malignant cells, the biopsy site would require complete subsequent removal (en bloc) and should be planned carefully to maximise the opportunity for limb salvage. Preoperative puncture biopsies were performed to determine the pathological classification of the tumours. Four cases were diagnosed as chordomas, three as chondrosarcomas, three as invasive giant cell tumours of the bone, two as Ewing’s sarcomas, and two as malignant neurinomas. All patients reported preoperative lumbosacral pain or constipation. The mean operative time was 6.54 hours (5.5–8.0 h), and the mean intra-operative blood loss was 2935 mL (1800–6500 mL). All patients were followed up for a mean of 62 months (24–108 months). Table 1 shows the patients’ characteristics. This study was approved by the institutional review board of our hospital, and the patients provided informed consent to publish.

**Table 1:** Demographic data of patients

Case no.	Sex	Age, years	Pathological type	Tumour location	Surgical duration, h	Intra-operative blood loss, ml	Follow-up time, month
1	Male	43	Chondrosarcoma	S1–S4	7.5	1950	46
2	Female	14	Malignant neurinoma	S1–S3	6.0	2500	74
3	Male	28	Chordoma	S1–S5	6.5	2000	62
4	Female	39	Invasive giant cell tumour of bone	S1–S2	7.0	3500	63
5	Male	58	Chondrosarcoma	S1–S4	6.5	2600	58
6	Male	42	Chordoma	S1–S4	4.5	1800	24
7	Male	53	Invasive giant cell tumour of bone	S1–S3	5.5	3500	47
8	Male	50	Chondrosarcoma	S1–S2	7.5	2050	68
9	Female	44	Chordoma	S1–S5	8.0	4200	76
10	Female	38	Invasive giant cell tumour of bone	S1–S3	7.0	6500	82
11	Male	53	Chordoma	S1–S4	6.0	2090	108
12	Female	16	Ewing’s sarcoma	S1–S3	6.5	2300	52
13	Male	63	Malignant neurinoma	S1–S2	6.0	2500	48
14	Female	36	Ewing’s sarcoma	S1–S3	7.0	3600	60

### Pre-operative planning

Pre-operative arrangements were made for laboratory examinations of the whole blood, haematocrit, plasma, and other parameters. Pre-operative selective vascular embolisation was used to block the tumour’s blood supply. A ureteral catheter was placed using a cystoscope to facilitate intra-operative identification of the ureter and to avoid unintended damage. An adequate pre-operative enema was performed to reduce any interference intraoperatively. Artificial blood vessels were prepared, and anastomosis was performed if a blood vessel was damaged.

### Surgical method

**Fig. 1:**
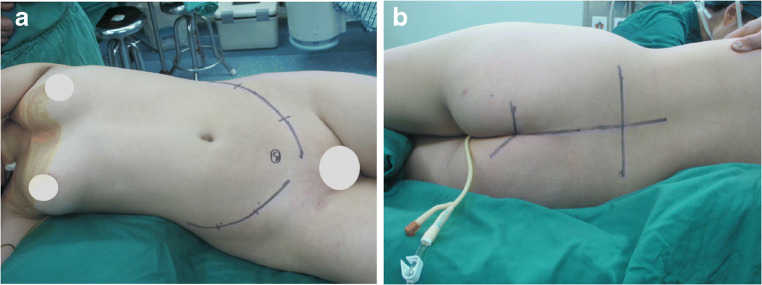
Surgical position of the combined antero-posterior surgical approach. **a** The anterior abdomen is accessed through a bilateral V-shaped incision through the extraperitoneal space. **b** Posterior incision via a transverse H-shaped incision

A combined antero-posterior surgical approach was used after the induction of satisfactory anaesthesia with the patient in a side-lying floating position. The anterior abdomen was accessed with bilateral V-shaped incisions through the extraperitoneal space while protecting the iliac blood vessels, intestinal organs, ureters, and other important structures (Fig. 1a). The bilateral iliac vessels were dissociated, and small branches were ligated. The soft tissue in front of the tumour was separated, and the intervertebral disc between L5 and S1 was removed as much as possible. The sciatic nerves and the upper margins of the sacroiliac joint were separated. The posterior incision was made as a transverse H-shaped incision (Fig. 1b), and the deep fascia was incised to reach the sacrospinalis, which was subsequently dissociated. The dorsal side of the sacrococcyx, bilateral sacroiliac joints, part of the iliac crest, and the L5 spinous processes were then exposed. The sacrospinous, sacrotuberous, and coccyx ligaments were removed, and the rectum was exposed. The space between the rectum and sacrum was filled with gauze, and the rectum was pushed forward, ensuring that the intestinal wall was not damaged during separation. The sacral spinous process was excised, and the sacral canal, dural sac, and sacral nerves were exposed. The bilateral L5 nerve roots were carefully separated and retracted. Following this, the bilateral iliac bones were sawed off on the lateral side of the sacroiliac joint, and the sacrum was completely resected. Bilateral pedicle screws were placed at L4 and L5, and two screws were placed on both iliac crests. A bone allograft of appropriate length was placed between the bilateral iliac osteotomy surfaces. Lumbosacral stability was restored using pre-bent metal bars and transverse connecting bars. The incision was closed after placing drainage tubes.

### Post-operative treatment

Antibiotic treatment was administered post-operatively, and an inflatable leg pump was used to prevent venous thrombosis in the lower limbs. The drainage tube was removed when the daily drainage volume was <50 mL. The indwelling time of the catheter was determined based on whether the patient could urinate. If the patient was unable to urinate, the indwelling catheter was kept in place and bladder function exercises were continued. At 8 weeks postoperatively, the patients were instructed to stand up using crutches and perform progressive lower limb walking exercises.

### Follow-up plan and evaluation of efficacy

The patients had to undergo regular post-operative outpatient follow-up visits during the third, sixth and 12th month post-operatively for radiographic pelvic re-examination. After one year, the pelvic radiograph was reviewed once every six months; after three years, it was reviewed once a year. The visual analogue scale (VAS) and the Musculoskeletal Tumor Society (MSTS) scores were used to assess the degree of pain and lower limb function, respectively. Tumour recurrence, loosening of internal fixations, and bone graft healing were evaluated from the radiographs.

## Results

No patient died in the peri-operative period. At the last follow-up consult, ten patients were continuously disease-free, three patients were alive with disease, and one patient died of disease due to lung metastasis. While tumour recurrence occurred in three patients, none of them had distant metastasis and they remained continuously disease-free. However, there was no indication for reoperation, and only local radiotherapy was given for these patients.

At the last follow-up consult, the MSTS scores ranged from 6 to 28 (20.00–93.33%, 6/30–28/30), with an average score of 20 (66.67%, 20/30). At the time of their last clinical evaluation, seven of the 14 patients could walk independently in public, five could only walk at home using a walking aid, one could only lie down because of disease progression, and one could only sit or stand for a short time due to spinopelvic instability.

Thirteen patients developed post-operative complications including skin necrosis, screw loosening, and connecting rod fracture, amongst others. Skin necrosis at the surgical incision site was observed in two cases, but the wound healed after debridement. The bladder sphincter function in one patient was essentially normal, while twelve patients noted urgency with urinary incontinence. Stress incontinence can occur when urine volume is high or when urine is retained. After two to six months of autonomic bladder function exercises (mean, 4 months), these patients were able to either compress their bladder or urinate on their own. Most patients noted bowel urgency under increased pressure. These patients were instructed to perform defecation exercises, follow diet control, and partake in medication-assisted therapy. All patients who followed these instructions were able to defecate by themselves eventually, while the rest defecated by squeezing their lower abdomen regularly. Neuropathic pain was observed in eight patients, and the VAS scores ranged between one and four after six months of treatment. Post-operative screw loosening occurred in three patients, while connection rod fracture occurred in two; however, these two patients refused revision surgery. Post-operatively, eight patients noted bilateral sciatic nerve symptoms including plantar flexion movement disorders and hypoaesthesia of the skin at the base of the foot; these patients could walk after ankle brace fixation.

**Fig. 2:**
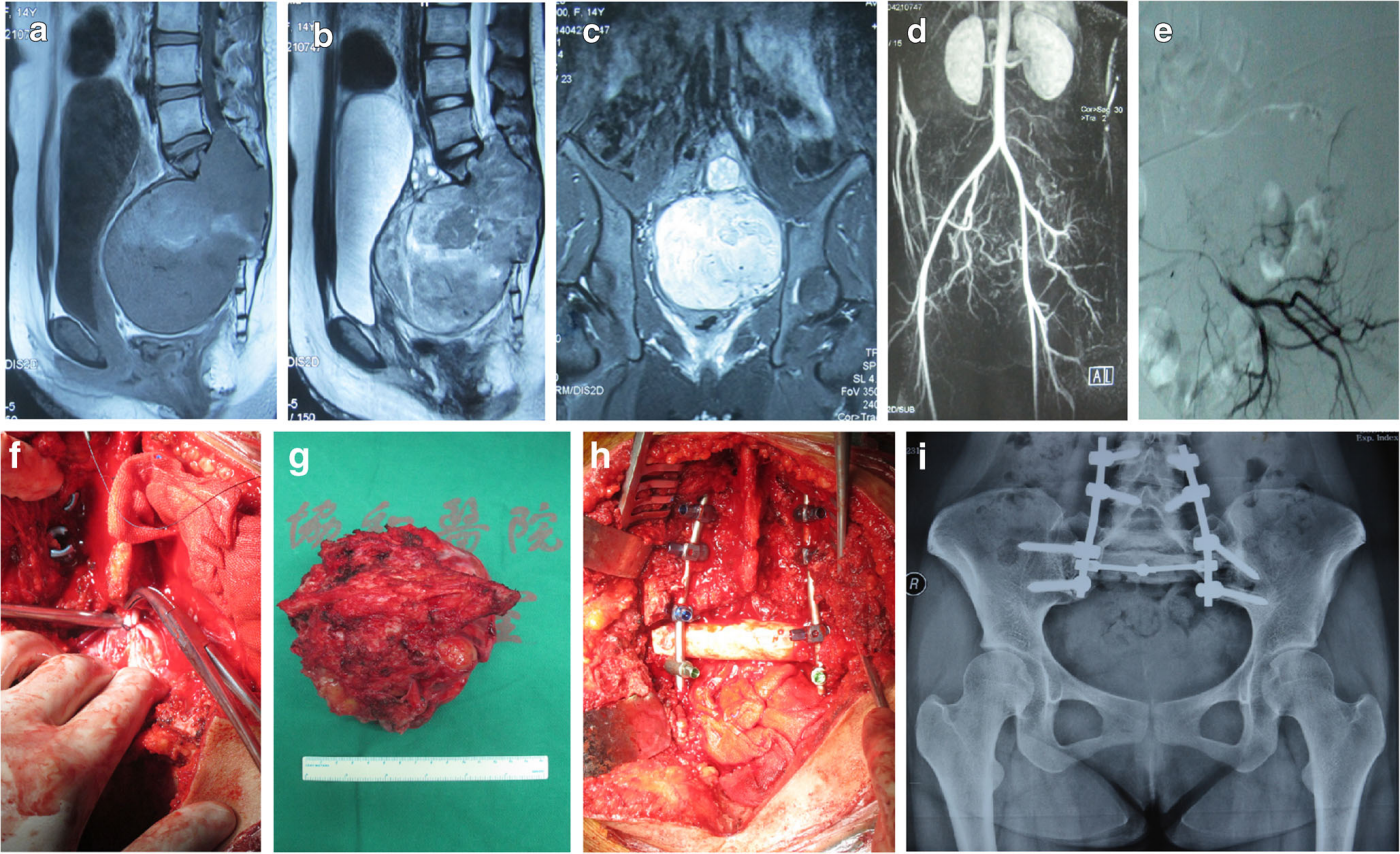
A 14-year-old female patient with a malignant schwannoma. **a** T1-WI MRI showing mixed iso-low signals. **b** T2-WI MRI showing mixed high signals. **c** The coronal view of the MRI showing the filling of the pelvic cavity by the tumour. **d** Angiography showing abundant blood supply in the tumour. **e** Selective vascular embolisation is used to control surgical bleeding. **f** Ligation of the dura. **g** General view of the specimen after total sacrectomy. **h** Reconstruction of ilio-lumbar stability using a large segment allograft and spinal internal fixation system. **i** Three-year post-operative radiograph showing good fusion of the allograft, stable internal fixation, and no loosening or fracture of screws and connecting rods. WI, weighted image; MRI, magnetic resonance imaging

**Fig. 3:**
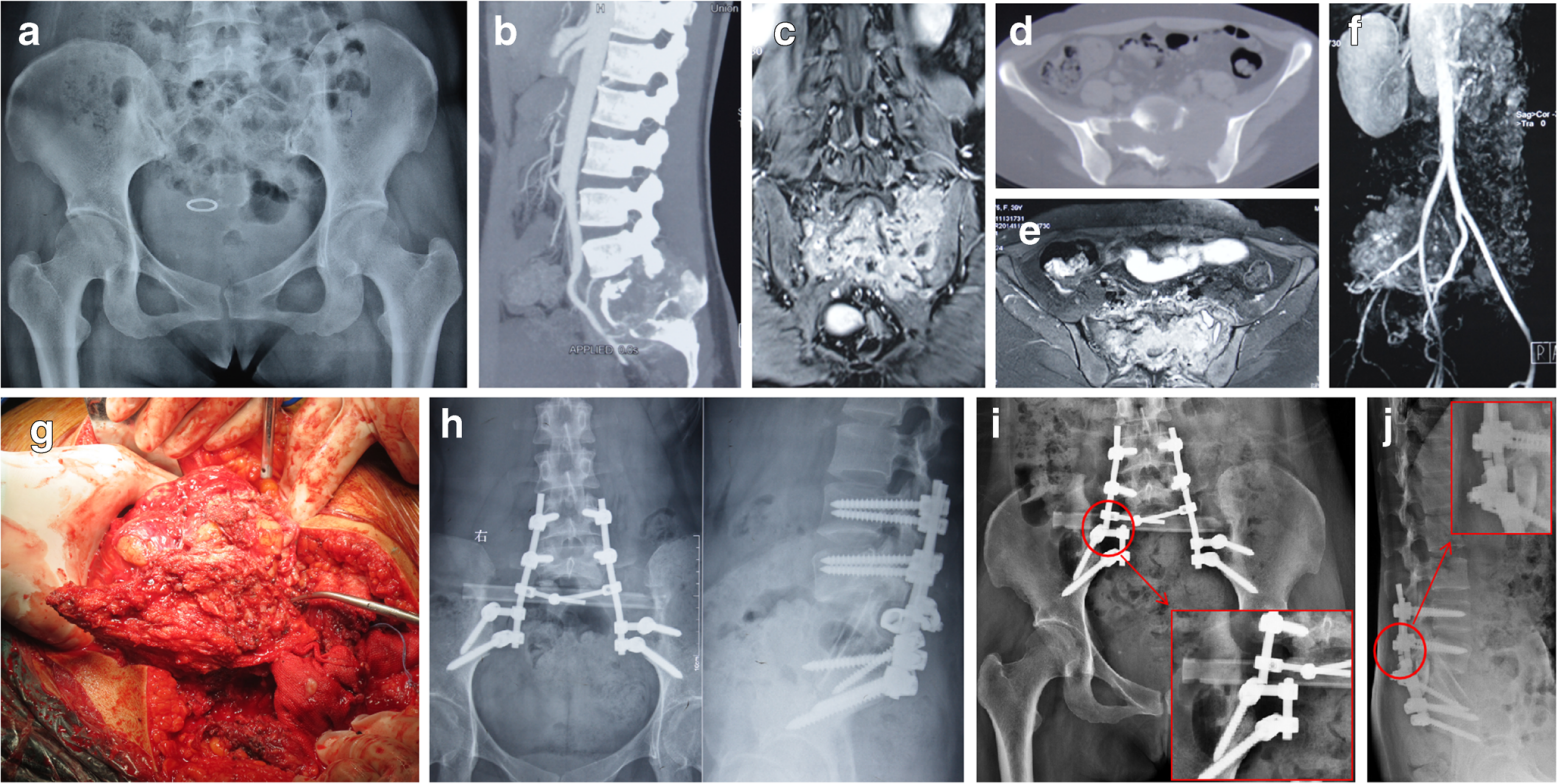
A 39-year-old female patient with an invasive giant cell tumour of the bone. **a** Pre-operative radiograph showing osteolytic destruction of the sacrum. **b**, **d** Pre-operative CT showing osteolytic bone destruction in the S1–S3 regions, with multilocular pores and bone crests inside. **c**, **e** T2-WI MRI shows mixed high signals and low signals in the bone crests. **f** Angiography showing abundant blood supply of the tumour. **g** Total sacrectomy. **h** One-year post-operative radiograph showing good fusion of the allograft, stable internal fixation, and no loosening or fracture of screws and connecting rods. **i**, **j** Two-year postoperative radiograph showing fracture of the right connecting rod. CT, computed tomography; WI, weighted image; MRI, magnetic resonance imaging

Table 2 shows the oncologic and functional outcomes of the patients. Two typical cases are represented in Figs. 2 and 3, respectively.

**Table 2:** Oncologic and functional outcome of patients

Case no.	Status	Tumour recurrence	Distant metastasis	Musculoskeletal Tumor Society score	Ambulatory status	Post-operative complications
1	Continuous disease-free	No	No	24	Public, independent walking	Neuropathic pain, sciatic nerve injury, dysuria
2	Continuous disease-free	No	No	28	Public, independent walking	Neuropathic pain, sciatic nerve injury, dysuria
3	Continuous disease-free	Yes	No	18	Household walking	Dysuria
4	Continuous disease-free	No	No	18	Household walking	Sciatic nerve injury, dysuria
5	Continuous disease-free	Yes	No	24	Public, independent walking	Skin necrosis, neuropathic pain, dysuria
6	Continuous disease-free	No	No	20	Household walking	Neuropathic pain, sciatic nerve injury, dysuria
7	Continuous disease-free	No	No	22	Public, independent walking	Screw loosening, sciatic nerve injury, dysuria
8	Alive with disease	No	No	24	Public, independent walking	Dysuria
9	Alive with disease	No	No	26	Public, independent walking	Neuropathic pain, sciatic nerve injury, dysuria
10	Continuous disease-free	No	No	16	Household walking	Neuropathic pain, sciatic nerve injury, urinary incontinence
11	Continuous disease-free	No	No	24	Public, independent walking	No
12	Died of disease	No	Yes	18	Household walking	Skin necrosis, neuropathic pain, dysuria
13	Continuous disease-free	Yes	No	6	Only lie down	Screw loosening, connection rod fracture, neuropathic pain, sciatic nerve injury, dysuria
14	Alive with disease	No	No	12	Sit and short time stand	Screw loosening, connection rod fracture, dysuria

## Discussion

Tomita proposed the use of total sacrectomy for the treatment of sacral tumours [11]. Although most patients have permanent neurological deficits after tumour resection, extensive/adequate excision is the best treatment for sacral tumours to reduce the chance of local recurrence and prolong survival time [9, 10, 13]. The sacrococcygeal anatomical structure is complex and makes complete resection of the tumour difficult; furthermore, post-operative local recurrence rates are high [12]. Therefore, it is important to select an appropriate surgical approach, protect the nerves and blood vessels, avoid injury to the intestine or ureters, control the risk of bleeding, and restore lumbosacral stability [14].

The surgical approach chosen depends on the location of the tumour and its relationship with the surrounding structures [15]. Total sacrectomy with an anterior approach is convenient for separating important structures such as the anterior blood vessels and rectum. However, it is difficult to separate and amputate nerve roots and perform sacral osteotomy [16]. In total sacrectomy with a posterior approach, the sacral tumour should be separated from the rectum, lateral soft tissue, and ligaments in the same incision [17]. Consequently, with this approach, the pull and exposure times of the skin incision margins are significantly higher than those of the combined antero-posterior approach, as is the incidence of wound complications [18]. Moreover, using the posterior approach in exposing the anterior sacrum increases the incidence of rectal injury due to the limited space and exposed field available [19]. To achieve complete tumour resection, most scholars use the combined antero-posterior approach, which can safely separate the rectum and blood vessels in front of the sacrum and allows closure of the posterior incision with a rectus or omental flap [20, 21].

The sacrum and its surrounding tissues are mainly supplied by the internal iliac artery and the middle sacral artery [22]. The accompanying veins and the sacral vertebral veins form a venous plexus in front of the sacrum with abundant collateral circulation [22]; this provides the tumours with a rich blood supply and abundant anastomosis, thereby predisposing them to bleed rapidly on injury [20]. Compression of the common iliac vein, internal iliac vein, and anterior sacral venous plexus by the tumour creates venous stasis, which also predisposes them to intra-operative rupture and bleeding [23, 24]. Pre-operative selective vascular embolisation has shown good control of surgical bleeding [25]. In this study, we used a gelatine sponge as an embolising agent and operated within 24 hours after embolisation. The mean operative time was 5.5 hours (4.5–8.0 h), the mean intra-operative blood loss was 3500 mL (1800–6500 mL), and no patient died in the peri-operative period. Hence, we believe that preoperative embolisation of the main and collateral arteries supplying the sacral tumour reduces the risk of intra-operative bleeding.

Total sacrectomy causes instability in the pelvis and spine because of the involvement of the sacroiliac joint; thus, it is important that ilio-lumbar stability is restored in these patients [26]. Although many reconstruction methods reported in the literature provide immediate post-operative stability, most researchers recommend bed rest after total sacrectomy; this allows the wound to stabilise and a scar to gradually form before the patient begins standing up [27]. Currently, lumbosacral and ilio-lumbar reconstructions are performed after sacral tumour resection, and a spinal internal fixation system is most often used [28]. Reconstruction methods structurally fall into two categories: the use of an iliac screw and screw rod to support the lumbar spine directly on the iliac crest, and the use of bone grafts or internal fixation to connect the iliac bones bilaterally to restore the posterior pelvic ring. In the latter method, stress transmission from the lumbar spine to the iliac crest is restored by connecting the posterior pelvic ring with a transverse metal rod through a nail rod system [29, 30].

After fixation of the lumbar pelvis, the torque lengthens, the lumbosacral stress and shear forces increase, and stress becomes concentrated in the metal internal fixation; all these factors predispose the metal implant to fatigue fractures in the long term [31]. All cases of metal rod fractures in this group occurred with bilateral single-rod fixation, indicating that this complication may be related to the low number of metal rods. Comparisons of different methods of lumbosacral reconstruction after total sacral resection with cadaver specimens have shown that unilateral two-rod fixation was superior to unilateral single-rod fixation under the pre-condition of restoring the pelvic ring structure; this may significantly reduce the amount of stress at the screw-bone interface [32]. A large distance between the waist and the iliac bones is also a risk factor for fractures [7]. A larger distance increases the range of lumbar spine motion and concentrates the stress on the metal connecting rod, eventually leading to metal rod fractures [33]. Therefore, we believe that in ilio-lumbar reconstruction, the distance between the lumbar screw and the iliac screw should be shortened as much as possible. Generally, the lumbar screw should be fixed on the fourth and fifth vertebral bodies [6]. However, no systematic study has been conducted to determine whether increasing the number of screws reduces fixation failure. Biological reconstruction is essential for long-term lumbosacral stability, and structural bone grafting can be performed using different bone-grafting materials, including autogenous fibula and allografts [6, 33]. We used a large allograft segment combined with an internal fixation system to pressurise the bone to improve graft healing. However, there are issues associated with reconstruction of the posterior pelvic ring and ensuring anterior column stability. The opening force of the posterior pelvic ring is very strong, and iliac screw fixation cannot counteract this opening force; this results in screw loosening, displacement, and posterior ring opening [26].

In addition to stability reconstruction, reconstruction of soft tissue should not be ignored [7]. After excision of sacral tumours, a large local cavity will remain that is covered only by a skin flap. Both embolisation and ligation of the internal iliac vessels, as well as surgical operations, can affect the blood supply of the flap, leading to post-operative flap necrosis or infection [34]. For patients with local soft tissue defects, creating a gluteus maximus muscle flap is indicated [8]. After sacral resection, the original ligaments and muscles lose their bony attachment points leading to post-operative pelvic organ prolapse [7, 8]. In our opinion, when suturing wounds, stable pelvic floor structures, such as the sacrospinous ligament, should be tightly sutured to the surrounding residual tissues as much as possible to prevent post-operative complications.

There are several technical difficulties that may be encountered during total sacrectomy with a combined antero-posterior surgical approach. First, in terms of bleeding control, our experience is that, for large presacral soft tissue masses, the anatomical relationship between the tumour and the blood vessels (the vena cava and internal iliac vein) should be determined pre-operatively by angiography. Second, reconstruction of lumbar iliac stability after total sacral resection has always been a difficult and important problem. Due to the complexity of the anatomical structure of the pelvis and its underlying mechanisms of stability, a reasonable reconstruction method needs further study. Short-term post-operative stability depends on metallic implants, while long-term post-operative stability is achieved by biological reconstruction of bone graft fusions. Thus, early fixation can create a more stable environment for bone grafting to ensure bone fusion, and late fixation can promote stress transmission without predisposing the metal implant to fatigue fractures. Finally, we also found a higher incidence of post-operative complications. Under the current reconstruction method, screw loosening and displacement, as well as fracture of the metal connecting rod, are the most common problems in the construction of a nail rod system. We think these may be associated with excessive torsion in the lumbar and iliac regions, as well as stress concentration, insufficient number of metal joints, improper fixation, non-fusion of bone grafts, and excessive body mass index of patients [35].

This study had a limited sample size and lacked an appropriate control group; therefore, we believe that studies with larger sample sizes, appropriate control groups, and longer follow-up period are necessary. Furthermore, studies that analyse the changes in the biomechanical state of the spine caused by internal fixation devices can provide more clinical evidence for optimising treatment options.

## Conclusion

Although total sacrectomy is a risky operation, complete tumour excision and reconstruction of the ilio-lumbar stability can ensure success. Better intra-operative bleeding control, proper preservation of the cauda equina nerve functions, and reconstruction of the pelvic ring are key considerations in surgical planning. Total sacrectomy and reconstruction are associated with several complications, and their efficacy still needs evaluation; hence, surgical indications should be carefully assessed.
